# Predicting Acute Brain Injury in Venoarterial Extracorporeal Membrane Oxygenation Patients with Tree-Based Machine Learning: Analysis of the Extracorporeal Life Support Organization Registry

**DOI:** 10.21203/rs.3.rs-3848514/v1

**Published:** 2024-01-11

**Authors:** Andrew Kalra, Preetham Bachina, Benjamin L. Shou, Jaeho Hwang, Meylakh Barshay, Shreyas Kulkarni, Isaac Sears, Carsten Eickhoff, Christian A. Bermudez, Daniel Brodie, Corey E. Ventetuolo, Bo Soo Kim, Glenn J. R. Whitman, Adeel Abbasi, Sung-Min Cho

**Affiliations:** Johns Hopkins University School of Medicine; Johns Hopkins University School of Medicine; Johns Hopkins University School of Medicine; Johns Hopkins University School of Medicine; Warren Alpert Medical School of Brown University; Warren Alpert Medical School of Brown University; Warren Alpert Medical School of Brown University; University of Tübingen; Perelman School of Medicine at the University of Pennsylvania, Philadelphia; Johns Hopkins University School of Medicine; Warren Alpert Medical School of Brown University; Johns Hopkins University School of Medicine; Johns Hopkins University School of Medicine; Warren Alpert Medical School of Brown University; Johns Hopkins University School of Medicine

**Keywords:** machine learning, extracorporeal membrane oxygenation, acute brain injury, Extracorporeal Life Support Organization, neurological complications

## Abstract

**Objective:**

To determine if machine learning (ML) can predict acute brain injury (ABI) and identify modifiable risk factors for ABI in venoarterial extracorporeal membrane oxygenation (VA-ECMO) patients.

**Design:**

Retrospective cohort study of the Extracorporeal Life Support Organization (ELSO) Registry (2009–2021).

**Setting:**

International, multicenter registry study of 676 ECMO centers.

**Patients:**

Adults (≥18 years) supported with VA-ECMO or extracorporeal cardiopulmonary resuscitation (ECPR).

**Interventions:**

None.

**Measurements and Main Results:**

Our primary outcome was ABI: central nervous system (CNS) ischemia, intracranial hemorrhage (ICH), brain death, and seizures. We utilized Random Forest, CatBoost, LightGBM and XGBoost ML algorithms (10-fold leave-one-out cross-validation) to predict and identify features most important for ABI. We extracted 65 total features: demographics, pre-ECMO/on-ECMO laboratory values, and pre-ECMO/on-ECMO settings.

Of 35,855 VA-ECMO (non-ECPR) patients (median age=57.8 years, 66% male), 7.7% (n=2,769) experienced ABI. In VA-ECMO (non-ECPR), the area under the receiver-operator characteristics curves (AUC-ROC) to predict ABI, CNS ischemia, and ICH was 0.67, 0.67, and 0.62, respectively. The true positive, true negative, false positive, false negative, positive, and negative predictive values were 33%, 88%, 12%, 67%, 18%, and 94%, respectively for ABI. Longer ECMO duration, higher 24h ECMO pump flow, and higher on-ECMO PaO_2_ were associated with ABI.

Of 10,775 ECPR patients (median age=57.1 years, 68% male), 16.5% (n=1,787) experienced ABI. The AUC-ROC for ABI, CNS ischemia, and ICH was 0.72, 0.73, and 0.69, respectively. The true positive, true negative, false positive, false negative, positive, and negative predictive values were 61%, 70%, 30%, 39%, 29% and 90%, respectively, for ABI. Longer ECMO duration, younger age, and higher 24h ECMO pump flow were associated with ABI.

**Conclusions:**

This is the largest study predicting neurological complications on sufficiently powered international ECMO cohorts. Longer ECMO duration and higher 24h pump flow were associated with ABI in both non-ECPR and ECPR VA-ECMO.

## Introduction

Extracorporeal membrane oxygenation (ECMO) is increasingly used for cardiopulmonary support.(1) Acute brain injury (ABI), which includes central nervous system (CNS) ischemia, intracranial hemorrhage (ICH) and hypoxic-ischemic brain injury, (HIBI) is reported to occur in up to 20% of adult venoarterial (VA)-ECMO patients(2) in the Extracorporeal Life Support Organization (ELSO) Registry. Furthermore, this rate is as high as 33% in VA-ECMO patients using noninvasive multimodal neuromonitoring at a single institution.(3) With greater ECMO usage and more cases of ABI, accurately predicting ABI with modifiable risk factors such as hyperoxia(4), low pulse pressure (PP)(5, 6), and hypercarbia(7) is important to lessen its occurrence.

In VA-ECMO, there have been several scoring systems developed to predict survival outcomes,(8–15) but their generalizability is limited as they stem from single-center studies, are focused in a specific subset of patients (e.g., only cardiogenic shock), and were created from logistic regression. Machine learning (ML) leverages big data to explore patterns and interactions without explicit programming from humans, thus offering distinct advantages to traditional regression.(16) Furthermore, coupled with the large sample size of the ELSO Registry, ML may be the most promising technique to adequately synthesize demographic and laboratory information to effectively predict ABI.(17) Additionally, identifying variables in the ML model that impact clinical outcomes will inform ECMO clinicians for mitigation of key risk factors for ABI.

Current literature applying ML to predict outcomes in ECMO patients is sparse and primarily focused on non-neurological outcomes such as thrombosis/hemorrhage and mortality.(18–20) An ELSO Registry analysis of VA-ECMO patients (n = 23,812) demonstrated ML yielded better prediction for in-hospital mortality (AUC-ROC = 0.80) versus the SAVE score (AUC-ROC = 0.61).(19) This study demonstrated the power of ML when applied to the ELSO Registry, and provided the impetus for this study designed to test the capability of ML to predict ABI.

Herein, we aimed to leverage ML to predict ABI in a large international cohort (the ELSO Registry) of ECMO patients.

## Methods

### Study design and population

The Johns Hopkins Hospital Institutional Review Board approved this retrospective observational study (IRB00216321) with a waiver of informed consent. “Retrospective Analysis of Outcomes of Patients on Extracorporeal Membrane Oxygenation” is the study title. All procedures were followed in accordance with the Helsinki Declaration of 1975 and the ethical standards of the responsible committee on human experimentation (institutional or regional). The ELSO Registry is an international multicenter database from over several hundred ECMO centers worldwide.(21) It collects clinical characteristics and demographics, pre-ECMO and on-ECMO laboratory values such as arterial blood gas (ABG), on-ECMO complications, and outcomes like in-hospital mortality.^16^ Comorbidity information was captured using the *International Classification of Diseases, 10th Revision (ICD-10)* codes.

We included patients who were 1) 18 years of age or older; and 2) supported with VA-ECMO for extracorporeal cardiopulmonary resuscitation (ECPR) and non-ECPR indications from 2009–2021. We excluded repeat ECMO runs within the same patient to avoid bias and complexity. VA-ECMO and ECPR cohorts were analyzed separately.

### Data collection

**Figure 1:**
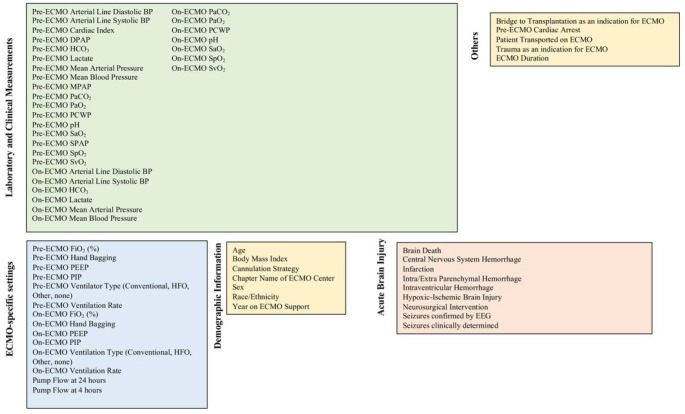
All 65 variables incorporated into our machine learning models including laboratory values, ECMO settings, demographics, other variables, and our primary outcome (acute brain injury). BP: blood pressure. CI: cardiac index. DBP: diastolic blood pressure. DPAP: diastolic pulmonary arterial pressure. ECMO: extracorporeal membrane oxygenation. EEG: electroencephalogram. FiO_2_: fraction of inspired oxygen. HFV: high frequency ventilator. MPAP: mean pulmonary arterial pressure. PaO_2_: partial pressure of oxygen. PaCO_2_: partial pressure of carbon dioxide. PCWP: pulmonary capillary wedge pressure. PEEP: positive-end expiratory pressure. PIP: peak inspiratory pressure. SPAP: systolic pulmonary arterial pressure. SaO_2_: arterial blood gas oxygen saturation. SpO_2_: peripheral oxygen saturation. SvO_2_: mixed venous oxygen saturation.

In total, 65 variables were collected (Fig. 1) for ML. The ELSO Registry collects ABG and hemodynamics pre-ECMO support and on-ECMO. Both pre-ECMO ventilator settings and ABGs were drawn within 6 hours of starting ECMO cannulation. If multiple ABGs existed within a specific period, the pre-ECMO ABG that was nearest to the start of ECMO cannulation was chosen. On-ECMO hemodynamic and ABG information were drawn closest to 24 hours of ECMO support. Values that were meant to be obtained simultaneously such as systolic and diastolic blood pressure and oxygen saturation by pulse oximetry and by arterial blood gas were abstracted by a trained ELSO data manager/abstracter from each center and were collected concurrently.

### Definitions

ABI was defined as the presence of infarction (ischemic stroke), diffuse ischemia (HIBI), intra/extra parenchymal hemorrhage, intraventricular hemorrhage, seizures determined by electroencephalograph or clinically, and neurosurgical intervention (examples include intracranial pressure monitor, external ventricular drain, and craniotomy) during ECMO support. CNS ischemia was defined as ischemic stroke (determined by ultrasound, computed tomography (CT), or magnetic resonance imaging (MRI))) and HIBI (determined by CT or MRI). ICH was defined as intra/extra parenchymal hemorrhage and intraventricular hemorrhage (both determined by CT or MRI). Definitions for other variables included in our analysis are in the **Supplemental Methods.**

### Outcomes

The primary outcome was the occurrence of ABI during ECMO support. Secondary outcomes included subtypes of ABI such as CNS ischemia and ICH.

### Statistical analysis

Continuous variables were represented as median with interquartile range. Categorical variables were presented as frequency with percentages. The Wilcoxon rank-sum and Pearson’s chi-square tests were utilized to compare continuous and categorical variables, respectively. Statistical significance was set at a p-value < 0.05.

### Data Pre-Processing

All categorical variables were one hot-encoded prior to running ML algorithms. Multiple imputation was used for missing data. All missing variables are shown in **Supplemental Table 1.**

### Machine Learning Algorithm and Pipeline

We examined the suitability of 4 ML algorithms in predicting ABI from the ELSO Registry containing variables from pre-ECMO support and during ECMO support: Random Forest, CatBoost, LightGBM and XGBoost. For each algorithm, we fine-tuned the hyperparameters and used a Bayesian optimization onto our dataset split randomly into training (70%) and test (30%) sets. Further details are noted in the **Supplemental Methods**.

### Feature Importance Scores in ML

To better understand how these ML models were constructed and to determine which variables were most important in predicting ABI, we analyzed which variables were of highest importance in correctly predicting ABI. Specifically, we examined the ranked feature importance in the best performing models, which discloses the contribution of each variables in the composition of the boosted decision trees within the model. Furthermore, Feature Importance Scores and Shapley Additive Explanations (SHAP) values depict the contribution of a variables on the predictions of the model (**Supplemental Methods)**. Both Feature Importance Scores and SHAP values add interpretability to the model framework and reveal pertinent clinical variables associated with ABI. All statistical analyses were performed using R Studio (R 4.1.2, www.r-project.org) and Python.

## Results

### VA-ECMO (non-ECPR)

**Figure 2:**
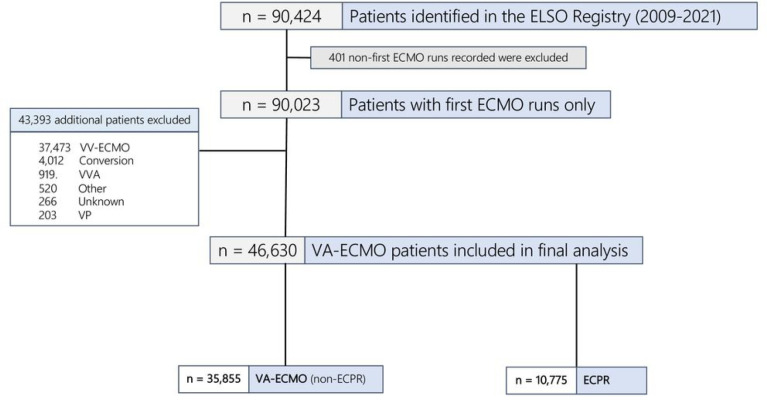
Flowchart of study cohort (VA-ECMO and ECPR patients) from the ELSO Registry in 2009–2020. ECMO = extracorporeal membrane oxygenation, VA = venoarterial, VV = venovenous, Conversion = VA -> VV or VV -> VA, ECPR = extracorporeal cardiopulmonary resuscitation, VVA = venovenoarterial, Other = mode not defined, VP = venopulmonary

Of 35,855 VA-ECMO (non-ECPR) patients, 2,769 (8%) had ABI (**Supplemental Table 2**, Fig. 2). The median age was 57.8 years (interquartile range, IQR:45.9–66.4) and 66% (n = 23,542) were male. The median duration of ECMO support was 4.3 days (IQR:2–7.7).

### Model Performance

**Figure 3:**
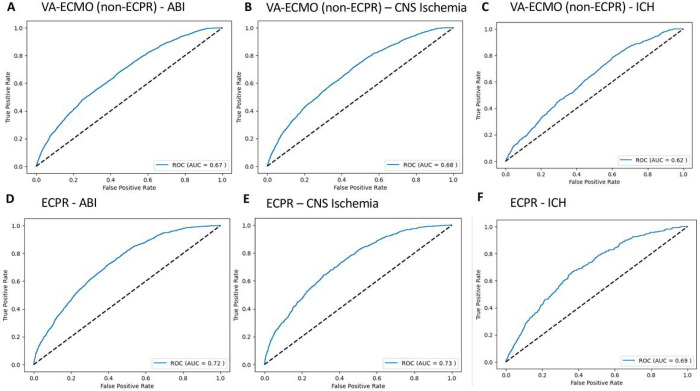
Receiver-operating characteristic curves for predicting **A)** acute brain injury (ABI), **B)** central nervous system (CNS) ischemia, and **C)** intracranial hemorrhage (ICH) in venoarterial extracorporeal membrane oxygenation (VA-ECMO) patients and for predicting **D)** ABI, **E)** CNS ischemia, and **F)** ICH in extracorporeal cardiopulmonary resuscitation (ECPR) patients.

Using the leave-one-out-cross-validation (LOOCV) 10-fold approach, for predicting ABI in VA-ECMO patients, the model achieved an AUC-ROC of 0.67 (Fig. 3A). The accuracy of the model was 83%. The true positive rate, true negative rate, false positive rate, and false negative rate were 33%, 88%, 12%, and 67%, respectively (Table 1). The PPV and NPV were 18% and 94%, respectively.

For predicting CNS ischemia, the model achieved an AUC-ROC of 0.67 (Fig. 3B). The accuracy of the model was 86%. The true positive rate, true negative rate, false positive rate, and false negative rate were 33%, 88%, 12%, and 67%, respectively. The PPV and NPV were 11% and 97%, respectively.

For ICH, the model achieved an AUC-ROC of 0.62 (Fig. 3C). The accuracy of the model was 97%. The true positive rate, true negative rate, false positive rate, and false negative rate were 5%, 99%, 1%, and 95%, respectively. The PPV and NPV were 8% and 98%, respectively.

### Feature Importance

**Figure 4:**
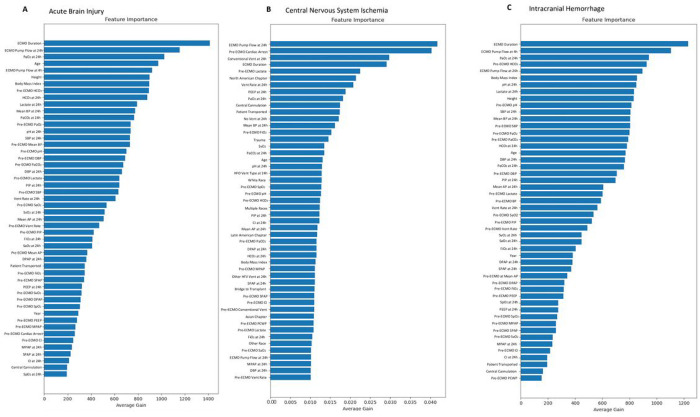
Feature Importance Scores for **A)** acute brain injury, **B)** central nervous system ischemia, and **C)** intracranial hemorrhage in VA-ECMO (non-ECPR) patients. AP: arterial pressure. BP: blood pressure. CI: cardiac index. DBP: diastolic blood pressure. DPAP: diastolic pulmonary arterial pressure. ECMO: extracorporeal membrane oxygenation. EEG: electroencephalogram. FiO_2_: fraction of inspired oxygen. HFV: high frequency ventilator. MPAP: mean pulmonary arterial pressure. PaO_2_: partial pressure of oxygen. PaCO_2_: partial pressure of carbon dioxide. PCWP: pulmonary capillary wedge pressure. PEEP: positive-end expiratory pressure. PIP: peak inspiratory pressure. SPAP: systolic pulmonary arterial pressure. SaO_2_: arterial blood gas oxygen saturation. SpO_2_: peripheral oxygen saturation. SvO_2_: mixed venous oxygen saturation. Vent: ventilator.

We identified the top 3 most important variables per Feature Importance Scores and depict the remaining variables (Fig. 4A, **Supplemental Fig. 1A, Supplemental Table 3**). The top 3 variables in predicting ABI were duration of ECMO support, ECMO pump flow rate at 24 hours, and on-ECMO PaO_2_.

The median ECMO duration was higher in patients with ABI versus patients without ABI (4.8 versus 4.3 days, p < 0.001). The median ECMO pump flow rate at 24 hours was higher in patients with ABI versus patients without ABI (4 versus 3.95 liters per minute, p < 0.001). The median on-ECMO PaO_2_ was higher in patients with ABI versus patients without ABI (162 versus 141 mmHg, p < 0.001). The top 3 variables in predicting CNS ischemia were ECMO pump flow rate at 24 hours, pre-ECMO cardiac arrest, and conventional ventilation at 24 hours of ECMO support (Fig. 4B, **Supplemental Fig. 1B, Supplemental Table 4**). The median ECMO pump flow rate at 24 hours was higher in patients with CNS ischemia versus patients without CNS ischemia (4 versus 3.95 liters per minute, p < 0.001). The prevalence of CNS ischemia in patients with pre-ECMO cardiac arrest was higher than patients without cardiac arrest (5.8% versus 3.3%, p < 0.001). The prevalence of CNS ischemia in patients with conventional venting at 24 hours of ECMO support was higher than patients without conventional venting at 24 hours of ECMO support (8.6% versus 2.7%, p < 0.001). The top 3 variables in predicting ICH were duration of ECMO support, ECMO pump flow rate at 4 hours, and on-ECMO PaO_2_ (Fig. 4C, **Supplemental Fig. 1C, Supplemental Table 5**). The median ECMO duration was higher in patients with ICH versus patients without ICH (6 versus 4.3 days, p < 0.001). The median ECMO pump flow rate at 4 hours was higher in patients with ICH versus patients without ICH (3.98 versus 3.82 liters per minute, p < 0.001). The median on-ECMO PaO_2_ was similar between patients with ICH versus patients without ICH (151 versus 142 mmHg, p = 0.27).

### ECPR

Of 10,775 ECPR patients, 1,787 (16.5%) had ABI (Fig. 1, **Supplemental Table 6)**. The median age of the ECPR cohort was 57.1 years (IQR:45.5–65.9) and 68% (n = 7,388) were male. The median duration of ECMO support was 2.63 days (IQR:0.88–5.33).

### Model Performance

For predicting ABI in ECPR patients, the model achieved an AUC-ROC of 0.72 (Fig. 3D). The accuracy of the model was 69%. The true positive rate, true negative rate, false positive rate, and false negative rate were 61%, 70%, 30%, and 39%, respectively (Table 2). The PPV and NPV were 29% and 90%, respectively.

For predicting CNS ischemia, the model achieved an AUC-ROC of 0.73 (Fig. 3E). The accuracy of the model was 81%. The true positive rate, true negative rate, false positive rate, and false negative rate were 41%, 85%, 15%, and 59%, respectively. The PPV and NPV were 18% and 95%, respectively.

For ICH, the model achieved an AUC-ROC of 0.69 (Fig. 3F). The accuracy of the model was 88%. The true positive rate, true negative rate, false positive rate, and false negative rate were 28%, 89%, 11%, and 72%, respectively. The PPV and NPV were 7% and 98%, respectively.

### Feature Importance

The top 3 variables for predicting ABI were duration of ECMO support, age, and ECMO pump flow rates at 24 hours and further details are depicted in the **Supplement (Supplemental Fig. 2, Supplemental Fig. 3, Supplemental Tables 7–9, Supplemental Results).**

## Discussion

This is the first ML study leveraging a large international database to predict ABI in ECMO patients, conveying novelty and generalizability of our study’s results. In 35,855 VA-ECMO (non-ECPR) and 10,775 ECPR patients, ML predicted ABI during ECMO support with an AUC-ROC of 0.67 and 0.70 in VA-ECMO (non-ECPR) and ECPR patients, respectively, while identifying risk factors for their occurrence.

### VA-ECMO vs. Venovenous (VV)-ECMO risk factors

ML uniquely identified longer duration of ECMO support, higher ECMO pump flow rate at 24 hours of ECMO support, and higher on-ECMO 24-hour PaO_2_ as the top 3 most important variables associated with ABI. In another study applying ML to predict ABI in VV-ECMO, (22)ECMO duration was also a top 3 most important variable for ABI; however, a longer duration of ECMO support was associated with lower risk of ABI in VV-ECMO patients while it was associated with a higher risk in VA-ECMO. As VV-ECMO patients have been shown to be cannulated longer than VA-ECMO patients,(23–25) the longer ECMO duration and lower risk of ABI associated may be attributed to the withdrawal of life-sustaining therapy for severely sick patients.(26, 27) Accordingly, this may have created a selection bias for patients who did undergo ABI and survived on ECMO support for longer. Furthermore, a higher ECMO pump flow rate and likely corresponding hemolysis(28) was uniquely important for ABI in VA-ECMO and ECPR, but not in VV-ECMO. This finding may reflect the different hemodynamic/physiological states(28, 29) and use/disuse of an aortic cannula(30) in VA-versus VV-ECMO populations and warrants further study. Although cross-sectionally the ECMO pump flow rates were small and may not be clinically meaningful, over the duration of ECMO support these differences may accrue to substantial differences. While pre-ECMO cardiac arrest is a known risk factor for CNS ischemia in ECPR patients,(2, 31) likely related to reperfusion injury and associated reactive oxygen species formation,(31, 32) we also note that this factor was highly important in VV-ECMO patients(33) which has not been previously reported. These comparisons suggest there are similar underlying but overall divergent risk factors between these populations, which necessitates further investigation with prospective observational studies. Finally, although PaCO_2_ and PP have been previously shown to be important factors for ABI in regression analyses using the ELSO Registry,(4, 6) they were not in the top 3 most important variables in ML for any population but remained within the top 20.

### Machine learning methodologies

We chose tree-based ML algorithms to predict ABI, which are becoming more commonly used in healthcare studies(34) as they provide an effective way to consider all different possible outcomes in a model. Furthermore, these tree-based ML models demonstrate high power, good accuracy, and provide interpretability to the models. (35) The primary difference between using Random Forest vs. gradient boosting tree methods is that Random Forest trees are constructed in an independent fashion while gradient boosting methods are created sequentially. Accordingly, Random Forest can determine their outputs without restriction of order while gradient boosting methods like XGBoost are restricted in a more fixed manner. There are also key differences within boosting methods: CatBoost may be most optimal for categorical data and can generate output more quickly than XGBoost or LightGBM. LightGBM demonstrates better accuracy and speed than XGBoost, but XGBoost is the more established ML algorithm, perhaps making it a very reliable ML tree-based method. Nevertheless, despite implementing these 4 powerful and innovative methods with oversampling to enhance statistical power, ML could still not accurately predict ABI in the ELSO Registry. This finding may suggest that the ELSO Registry does not capture causative variables for ABI over the entire duration of ECMO support which are needed to fully glean the insights and advantages of ML and ultimately identify modifiable risk factors for ABI. Finally, we note that while ML did not predict ABI with high accuracy, it did produce a strong NPV (94% and 90% for ABI in VA-ECMO and ECPR, respectively), suggesting our models’ true utility may lie in its high sensitivity and capability to rule out patients who truly did not have ABI.

### Lack of standardized neurological monitoring

Given the relatively mediocre performance in predicting ABI and its subtypes in both cohorts, we reveal certain limitations using a heterogenous, large dataset such as the ELSO Registry to predict ABI with ML. Specifically, unlike the institutional protocol at Johns Hopkins Hospital which uses standardized neurological monitoring with proven efficacy,(3) the protocols used to determine ABI across ECMO centers are neither standardized nor adjudicated/validated, and thus vary considerably. Accordingly, we only observed a 7.7% prevalence of ABI in VA-ECMO patients and 16.5% prevalence of ABI in ECPR patients within the ELSO Registry, which is considerably less than the prevalence of 33% at an experienced tertiary care ECMO center.(3) Therefore, this study calls for more sensitive and accurate detection of ABI and more granular collection of variables across ECMO centers. ABI can precede mortality and therefore identifying risk factors for ABI can help clinicians mitigate their occurrence and their associated mortality risk. In fact, a single-center study of 106 VA-ECMO and 68 VV-ECMO pediatric patients using ML to predict CNS ischemia and ICH showed a superior AUC-ROC (0.76) than ours with the ELSO Registry (0.67).(36) This result may not be surprising given the institution’s rigorous advanced neuroimaging technique to determine ABI and adjudication system by multiple clinicians. Accordingly, their prevalence of ABI (51% in VA/VV-ECMO mixed population) was much higher than ours with the ELSO Registry (7.7% in VA-ECMO and 16.5% in ECPR). Overall, an ELSO Registry addendum for neurological monitoring and imaging protocols may improve performance for ML to predict ABI.

### Limitations

The primary limitation of our analysis was the lack of standardized neurological monitoring protocols across ECMO centers and lack of ABI adjudication in the ELSO Registry. Correspondingly, we observed underdiagnoses of ABI. Likely due to low prevalence of ABI and lack of granularity in variables in the ELSO Registry, predicting this outcome using ML was challenging and resulted in suboptimal performance. Furthermore, the ELSO Registry lacks granularity with laboratory measurements as ABGs are only collected at a singular time point instead of multiple times throughout the ECMO run and were not collected at the same exact time point at each center. Finally, as this was a retrospective study, only associations could be determined.

## Conclusions

Using the largest database of ECMO patients globally, we present the first study to predict neurological outcomes on sufficiently powered international ECMO patient cohorts. Machine learning identified ECMO duration and higher pump flow rates as the most important risk factors for ABI in both VA-ECMO and ECPR cohorts. Overall, performance of ML models to predict ABI in VA-ECMO and ECPR patients was suboptimal likely due to lack of standardization of neuromonitoring protocols and data granularity in the ELSO Registry. This finding suggests that the detection and sensitivity rates for capturing ABI in ECMO patients across ECMO centers worldwide is substandard. Accordingly, standardized neurological monitoring and imaging protocols are urgently needed.

## Figures and Tables

**Table 1:** Model performance in venoarterial extracorporeal membrane oxygenation patients for predicting acute brain injury, central nervous system ischemia, and intracranial hemorrhage.

	AUC-ROC	Acc	TPR	TNR	FPR	FNR	PPV	NPV	Precision	Recall	F1	Brier Score
ABI	0.67	83%	33%	88%	12%	67%	18%	94%	0.18	0.34	0.24	0.175
CNS Ischemia	0.67	86%	33%	88%	12%	67%	11%	97%	0.11	0.34	0.16	0.21
ICH	0.62	97%	5%	99%	1%	95%	8%	98%	0.10	0.06	0.08	0.095

AUC-ROC: area under the receiver-operating characteristic curve. Acc: Accuracy. TPR: True Positive Rate. TNR: True Negative Rate. FPR: False Positive Rate. FNR: False Negative Rate. PPV: Positive Predictive Value. NPV: Negative Predictive Value. ABI: acute brain injury. CNS: central nervous system. ICH: intracranial hemorrhage.

**Table 2:** Model performance in extracorporeal cardiopulmonary resuscitation patients for predicting acute brain injury, central nervous system ischemia, and intracranial hemorrhage.

	AUC-ROC	Acc	TPR	TNR	FPR	FNR	PPV	NPV	Precision	Recall	F1	Brier Score
ABI	0.72	69%	61%	70%	30%	39%	29%	90%	0.28	0.61	0.39	0.14
CNS Ischemia	0.73	81%	41%	85%	15%	59%	18%	95%	0.18	0.41	0.25	0.14
ICH	0.69	88%	28%	89%	11%	72%	7%	98%	0.07	0.28	0.11	0.25

AUC-ROC: area under the receiver-operating characteristic curve. Acc: Accuracy. TPR: True Positive Rate. TNR: True Negative Rate. FPR: False Positive Rate. FNR: False Negative Rate. PPV: Positive Predictive Value. NPV: Negative Predictive Value. ABI: acute brain injury. CNS: central nervous system. ICH: intracranial hemorrhage.

## Key Points

### Question:

Can machine learning (ML) accurately predict acute brain injury (ABI) in venoarterial-extracorporeal membrane oxygenation (VA-ECMO) patients?

### Findings:

In a retrospective cohort study of 35,855 VA-ECMO and 10,775 extracorporeal cardiopulmonary resuscitation (ECPR) patients from the Extracorporeal Life Support Organization (ELSO) Registry (2009–2021), 7.7% (n=2,769) and 16.5% (n=1,787) experienced ABI, respectively. ML predicted ABI with an AUC-ROC of 0.67 and 0.72 in VA-ECMO and ECPR patients, respectively. Longer ECMO duration and higher pump flow were associated with ABI for both cohorts.

### Meaning:

Clinicians should closely monitor ECMO duration and pump flow rates for VA-ECMO/ECPR patients at risk of ABI.

## Abbreviations

ABG: arterial blood gas
ABI: acute brain injury
AUC-ROC: area under the receiver-operating characteristic curve
BMI: body mass index
CI: confidence interval
CNS: central nervous system
DBP: diastolic blood pressure
DPAP: diastolic pulmonary arterial pressure
ECMO: extracorporeal membrane oxygenation
ELSO: Extracorporeal Life Support Organization
ICH: intracranial hemorrhage
IQR: interquartile range
LOOCV: leave-one-out-cross-validation
ML: machine learning
MPAP: mean positive airway pressure
PaCO2: partial pressure of carbon dioxide
PaO2: partial pressure of oxygen
PCWP: positive capillary wedge pressure
PEEP: positive end-expiratory pressure
PIP: positive inspiratory pressure
PP: pulse pressure
SBP: systolic blood pressure
SD: standard deviation
SHAP: Shapley Additive Explanations
SPAP: systolic pulmonary arterial pressure
SvO2: venous oxygen saturation
VA: venoarterial
VV: venovenous
